# Cystitis1A lecture delivered before the Gross Medical Club, at Clarence, N. Y., January, 1904, and by request before the Surgical Section of the Buffalo Academy of Medicine, February 2, 1904.

**Published:** 1904-03

**Authors:** J. Henry Dowd

**Affiliations:** Buffalo, N. Y.


### Cystitis.[1]

By J. HENRY DOWD, M. D., Buffalo, N. Y.

IN presenting to you the subject of inflammation of the bladder, I hope I may be pardoned for purposely omitting much that can only be considered of value to the specialist. On the other hand, it will be my aim to bring to your notice salient points which, if carefully noted, should give you, as general practitioners, a good working basis in handling inflammation of this viscus.

### PHYSIOLOGY OF URINATION.

To readily understand inflammation when involving the region in and around the bladder, especially in the male, one must be conversant with the physiology of urination. Urine is retained in the male bladder by the action of two muscles, (*a*) the internal sphincter, involuntary in action, consisting of three or four muscular fibers and situated at the urethro-vesical opening, and (*b*) the cut-off or compressor, being powerful, voluntary in action and situated an inch and a half anterior to the internal, at the bulbo-prostatic junction. Under certain conditions these

1. A lecture delivered before the Gross Medical Club, at Clarence, N. Y., January, 1904, and by request before the Surgical Section of the Buffalo Academy of Medicine, February 2, 1904.

muscles may or may not act entirely under the control of the will. If the bladder is in normal condition, about one and a half to one and three-quarter hours after a previous evacuation the contained fluid therein exerts sufficient pressure to cause the internal sphincter to open, thus allowing urine to pass into the posterior urethra. When this part, which now becomes the true neck of the bladder, fills to a certain extent, the urination sensation occurs and evacuation follows.

It must be evident that as soon as urine trickles into this portion, if acute inflammation exists, nature will try to rid itself of the irritant. At the same time it is a fact that if inflammation exists here sufficiently to produce pus in any quantity this secretion will pass backward, mixing with the urine in the bladder and rendering it opaque. As the inflammation becomes more chronic sensibility is blunted to an extent that the interval between urinations becomes lengthened from two to two and a half and even to three hours. When pus is produced in great quantities, if the urine is passed in divided parts the last will be opaque, yet there may be no inflammation behind the internal sphincter. The same holds good as to the diagnosis of posterior urethritis from the two or three glass tests. Inflammation may exist but pus not being produced in sufficient quantities may adhere to the mucous membrane and be washed out with the first flow of urine, this being opaque while the secondary flow will be clear.

Although the bladder, with but little uneasiness to the patient, can be made a receptacle for 15 to 16 ounces of urine or other fluids, its average capacity should be considered as about 10 ounces.

### VARIETIES.

Generally speaking, but two varieties of cystitis exist: (*a*) superficial, or where the mucous membrane and submucous tissue is involved; and (*b*) interstitial, where the inflammation involves the muscular coats and interstitial tissue. Examples of these are, superficial—any inflammation of the bladder due to bacteria other than the tubercle bacilli; interstitial—inflammation due to the tubercle bacilli.

### FACTORS IN THE PRODUCTION OF CYSTITIS.

Here, as in inflammation of other parts, three factors are necessary: first, a weakened or damaged condition of the tissue; second, pyogenic bacteria; third, a medium for their development. In congestion we find a most suitable cause for weakening of the parts and, at the same time, there is no doubt but that with congestion the medium is produced from the different glands. The usual forms of bacteria found in cystitis and in order of frequency are: strepto- and staphylococci, colonbacilli, urobacillus liquefacius septicus, tubercle bacilli and gonococci.

### SYMPTOMS.

The symptoms, of course, vary according to the condition present and the cause. Generally speaking, three important symptoms are always present; first, frequency of urination; second, pain, and third, pus in the urine. Other symptoms at times, are hematuria, ammonical fermentation due to decomposition of urea by bacteria forming the carbonate of ammonia, albumin in the urine, and general systemic disturbances.

*Frequency.*—In acute conditions from any cause, desire is constantly present, but emptying the bladder will take place only about every 15 to 25 minutes during the day and, on an average, every hour during the night. As the condition becomes chronic the desire becomes less frequent until there may be an interval of an hour or more.

*Pain* is always a prominent symptom and in the acute condition is one of the most important. Although there is an almost constant tenesmus, there is pain of a sharp lancinating character always following the expulsion of the last few drops of urine. This, as with frequency, diminishes much in severity as the condition becomes chronic, except in the presence of stone, tumor, foreign body, ulcer or fissure at the urethro-vesicle opening, when it continues as the most prominent symptom.

*Pus.*—This product of degeneration is always present, varying in amount from a ground glass appearance (if free hemorrhage stained red) to one closely resembling cider.

*Hemorrhage.*—Blood is always present in the urine, the quantity varying, according to the condition present. Thus, in the chronic condition it may only be detected by the microscope, whereas in the acute condition the urine may be of a bright red color, and as the bladder contracts in the expulsion of the last few drops of urine, free bleeding generally takes place to the amount of from 30 to 60 drops.

*Ammonical Fermentation.*—This condition cannot be considered a symptom of cystitis, per se, unless the case is very old and in this condition there is always more or less residual urine, as in hypertrophied prostate, stricture, or the condition known as villous cystitis.

*Albumin.*—This substance cannot in any way be considered a symptom of cystitis although present in nearly every case. Omitting the kidney as a source, this product may be found (*a*) wherever there is hemorrhage; (*b*) where in the absence of hemorrhage, the inflammation has been of a severity that the deeper layers of epithelium have been or are being cast off, thus allowing a transudation of serum from the vessels. This being a well proved fact, it must be evident that albumin in the urine may have its origin anywhere from the meatus upward.

*General Symptoms.*—These depend entirely upon the condition present and the cause thereof. In acute cases the usual symptoms of inflammation are present, such as chilly sensations, fever and the like. In the chronic condition there may be no symptoms, other than a sallowness, irritability due to the effect on the nervous system caused by broken rest, pain and the like. When the condition is due to malignancy, as tuberculosis or cancer, the usual cachexia soon develops, together with a slight rise in temperature, loss of flesh and a feeling of lassitude.

*Differential Diagnosis.*—To be successful in the treatment of cystitis it is absolutely necessary that the true condition be ascertained. Special care must be exercised to differentiate from the acute posterior urethrites, for the reason that the advised treatment of inflammation involving the bladder acts as a powerful irritant when used in the above condition. Tuberculosis of the bladder is also considered for the same reason, for in this condition, except as a diagnostic agent, washing of the bladder and especially with silver is contraindicated.

### TREATMENT.

The treatment of cystitis should be carried on from the following standpoint: (1) the cause; (2) relief of distressing symptoms; (3) rendering the urine bland and unirritating; (4) control of inflammatory process; (5) general treatment.

The cause of cystitis must be ascertained in every case, yet I do not intend to advocate, but rather to firmly condemn searching an inflammed bladder for tumor, stone, or foreign bodies of any kind. With our present knowledge of the urine and a careful history of the case, I think I am safe in saying the above-named conditions are generally ascertainable. The same may be said of tuberculosis, and here the treatment so differs from other forms that where a positive diagnosis is possible, and instructions to follow carefully carried out, life may not only be prolonged but much suffering avoided. Where stricture, stone or foreign bodies are the causative agents, these should be removed at once, the remaining inflammatory conditions being treated as noted under headings. Having elucidated the more important general considerations, perhaps I can make myself more clearly understood by drawing upon imagination for a few cases not unusual in the clinical history and which, I will treat in accordance with the principles already laid down in this paper. It will not be denied that every case, though to a certain extent a law unto itself, in a general way, will be found to follow, for the most part, a well defined course.

### HYPOTHETICAL CASES.[1]

1. Diagnosis acute cystitis, no evidence of previous bladder trouble, stone, tuberculosis or the like, but patient has chronic gonorrhea, and has had sounds passed, one only 48 hours ago. *Treatment.*—After thorough evacuation of the bladder, a luke-warm solution of silver nitrate (distilled water must be used), 1-16,000 is placed in the bladder without a catheter by aid of the Ultzmann-Dowd syringe. The same treatment should hold good in all cases from acute infection whether from within or without, male or female (a sterilised glass catheter should be used in the female), excepting tuberculosis. In these conditions no medication seems necessary as the most beneficial results should be obtained in a few hours. For the sake of argument let us imagine this case becomes chronic and accompanied by the following symptoms: urination averaging every hour, slight pain, with bleeding, urine very acid and containing an excess of uric acid. I would prescribe the following:

| | | |
|---|---|---|
| ℞ | Ext. hyoscyami | gr. j. |
| | Ext. belladonnæ | gr. 1-6. |
| | Tr. cantharidis | m. j. |

M. ft. pil. No. 1.

This can be given every 3 or 4 hours. Although not absolutely necessary in every case, a most valuable adjunct is the addition of uriseptin in two teaspoonful doses. In this condition it may be of decided advantage to curtail the diet to a certain extent. All articles known to be rich in uric or oxalic acid producing elements should be interdicted. Irrigation should be continued about every third day, increasing the silver in strength as 1-12,000, 1-10,000, 1-8,000, and so on. The same condition when dealt with in a haphazard manner may reach a stage known as villous cystitis, or ulceration of the bladder; mucous membrane may develop, one of these being located at and involving the internal sphincter,—a condition which I have heretofore described as fissure of the bladder. These, of course, are quite rare conditions, and as I am dealing with the most common varieties, I will not pursue further this portion of the subject.

2. Patient aged 55, has a hypertrophied prostate, stricture, (10 F.), in the deep urethra, urine always contains a large

1. The hypothetical cases, although placed under three headings, will be found to include practically all inflammatory conditions of the bladder.

amount of pus, showing a chronic inflammation of the posterior urethra, bladder, and kidney pelvis. This condition has existed for years. Seventy-two hours ago, after severe wetting of the feet, retention results. In attempting to relieve himself, patient breaks the catheter, a small piece being left in the bladder. The passage of this instrument served temporarily to dilate the stricture, but in 24 hours acute cystitis develops. *Treatment.*—External urethrotomy with division of stricture, removal of foreign body, thorough washing of the bladder with silver nitrate solution 1-16,000 after which a tube (No. 30 F.) should be placed in the bladder for drainage. The following is prescribed:

℞ Ol. sant. albus .................................... m. 15.
Uriseptin ............................................ ʒ ij.

S.—Every three hours in a glass of water.

Once daily thorough washing of the bladder with formaldehyde solution 1-5,000, and every third day silver solution in increasing strength should be used. Diet should be as far as possible devoid of uric acid producing substances, yet not curtailed sufficiently to in any way weaken the patient. Drainage should be continued until the urine is fairly free of pus, when the tube can be withdrawn allowing the wound to heal, the bladder treatment being carried on from the meatus. At the same time the stricture must be looked after to prevent re-contraction.

3. Frequency three or four months, no instrumental interference. Suspicion of tuberculosis. Bladder capacity, 3 ounces. Silver solution 1-16,000, as the test agent causes severe pain, the patient telling afterward, this lasted for 20 to 24 hours.

Creosote valerinate, 3 minims, gradually increased to 10, three times daily. Rest being disturbed somewhat, the following pill will be found serviceable:

℞ Ext. hyoscyami .................................. gr. j.
Ext. belladonæ .................................. gr. 1-6.
Morph. sulph. .................................. gr. 1-10.

Given at bed time.

All instrumental interference and bladder washing should be avoided, remembering that these not only aggravate the condition, but cause increased suffering. Let us imagine this case comes second-handed and has been cystoscoped, catheterised, and in other ways maltreated, the patient is passing urine every 15 minutes with quite severe pain and some free bleeding. Here we have a case of what may be termed mixed infection, that is, not only tuberculosis, but simple inflammation due to direct infection. In this condition silver 1-16,000 used once or twice, although producing quite severe pains, has a most beneficial action. The diet here should be as far as possible free of substances known to cause highly acid or concentrated urine. Under no consideration should the bladder be opened, unless stone or foreign body be present, or as occasionally happens a few weeks before death, frequent with severe pain is most distressing, then draining will bring the end more peacefully.

378 FRANKLIN STREET.

